# Reducing Time from Pediatric Emergency Department Arrival to Dexamethasone Administration in Wheezing Patients

**DOI:** 10.1097/pq9.0000000000000738

**Published:** 2024-06-11

**Authors:** Andrew W. Kramer, Jessica Erlich, Karen Yaphockun, Daniel Roderick, Kristen Farkas, Amy W. Bryl, Kathryn H. Pade

**Affiliations:** From the Rady Children’s Hospital.

## Abstract

**Introduction:**

Asthma exacerbations are common presentations to pediatric emergency departments. Standard treatment for moderate-to-severe exacerbations includes administration of oral corticosteroids concurrently with bronchodilators. Early administration of corticosteroids has been shown to decrease emergency department length of stay (LOS) and hospitalizations. Our SMART aim was to reduce the time from arrival to oral corticosteroids (dexamethasone) administration in pediatric patients ≥2 years of age with an initial Pediatric Asthma Severity Score >6 from 60 to 30 minutes within 6 months.

**Methods:**

We used the model for improvement with collaboration between ED physicians, nursing, pharmacy, and respiratory therapists. Interventions included nursing education, dosage rounding in the electronic medical record, supplying triage with 1-mg tablets and a pill crusher, updates to an asthma nursing order set and pertinent chief complaints triggering nurses to document a Pediatric Asthma Severity Score in the electronic medical record and use the order set. Our primary outcome measure was the time from arrival to dexamethasone administration. Secondary outcome measures included ED LOS for discharged patients and admission rate. We used statistical process control to analyze changes in measures over time.

**Results:**

From October 2021 to March 2022, the average time for dexamethasone administration decreased from 59 to 38 minutes. ED LOS for discharged asthma exacerbation patients rose with overall ED LOS for all patients during the study period. There was no change in the admission rate.

**Conclusions:**

Using quality improvement methodology, we successfully decreased the time from ED arrival to administration of dexamethasone in asthma exacerbation patients from 59 to 38 minutes over 10 months.

## INTRODUCTION

Acute asthma exacerbations are common presentations to pediatric emergency departments (PEDs). It is estimated that 7% of all children aged 0–18 in the United States carry a diagnosis of asthma.^1–3^ Of these children, over 40% have at least one asthma exacerbation per year, leading to an estimated 1.6 million PED visits annually in the United States.^2,3^ These events contribute to PED overcrowding and result in missed time from school and work for patients and families^4^. Timely treatment of asthma exacerbations may provide relief to overcrowded PEDs and mitigate the resource burden of pediatric asthma exacerbations.

Systemic corticosteroids are standard therapy in treating asthma exacerbations due to antiinflammatory effects and synergistic action with bronchodilators.^5–8^ The oral corticosteroid peak onset is typically 4 hours, making early steroid administration in the emergency department (ED) imperative.^8–10^ Steroid administration within 1 hour of ED arrival is associated with decreased ED length of stay (LOS), admission rates, time to clinical improvement, and likelihood of ED return.^11–13^ Nonetheless, timely administration of steroids poses a challenge for high-volume PEDs, and steroid delays are common. Delays in steroid administration have been associated with higher ED patient volume, longer ED wait times, and presentation to the ED at certain high-volume times of the day.^14,15^

Although some efforts have been made to implement EMS protocols that allow for the prehospital administration of corticosteroids,^16^ the majority of patients presenting with asthma exacerbations receive these medications in-hospital. Standing orders, which allow nurses to initiate treatment before physician assessment, have become increasingly popular in PEDs and can reduce LOS.^17^ These systems have been successfully used in PEDs for steroid administration to patients with asthma and are associated with lower admission rates and shorter ED LOS.^18,19^

In our tertiary pediatric emergency care center, a need to reduce the time from ED arrival to corticosteroid administration in patients presenting with asthma exacerbation was identified. Oral dexamethasone is the preferred steroid in our PED, given its similar efficacy, longer half-life, and higher rates of postdischarge adherence when compared with other oral corticosteroids.^20^

We sought to implement a nurse-driven system for administering oral dexamethasone in our PED triage area, aiming to improve outcomes for patients with asthma presenting in exacerbation. Our specific goal was to reduce the time from ED arrival to dexamethasone administration from 60 to 30 minutes over 6 months in patients aged 2–18 years presenting with asthma exacerbation.

## METHODS

### Setting

This quality improvement (QI) initiative was conducted in a freestanding urban tertiary pediatric ED with an average volume of 100,000 patients annually. The study population consisted of patients aged 2–18 years who presented with a clinical asthma exacerbation (a history of asthma with a chief complaint of “asthma exacerbation,” “wheezing,” or “shortness of breath”) and scored ≥7 on the Pediatric Asthma Severity Score scale (moderate-to-severe exacerbation).^21^ This Pediatric Asthma Severity Score score was chosen as an exacerbation of that severity, which generally receives a higher emergency severity index and is seen in our main ED rather than a fast-track area. The study excluded patients with first-time wheeze or co-morbid cardiopulmonary disease and patients who had received oral steroids within 48 hours of ED presentation.

## PLANNING

The study was initiated in July 2021 in collaboration with a team composed of physicians, nurses, respiratory therapists, informaticists, and pharmacists. In addition, a multidisciplinary ED QI team that included PEM physicians, ED nurses, and ED respiratory therapists was assembled. An institutional review board reviewed and approved the project. Squire 2.0 guidelines^22^ were considered during planning and reporting.

**Fig. 1.**
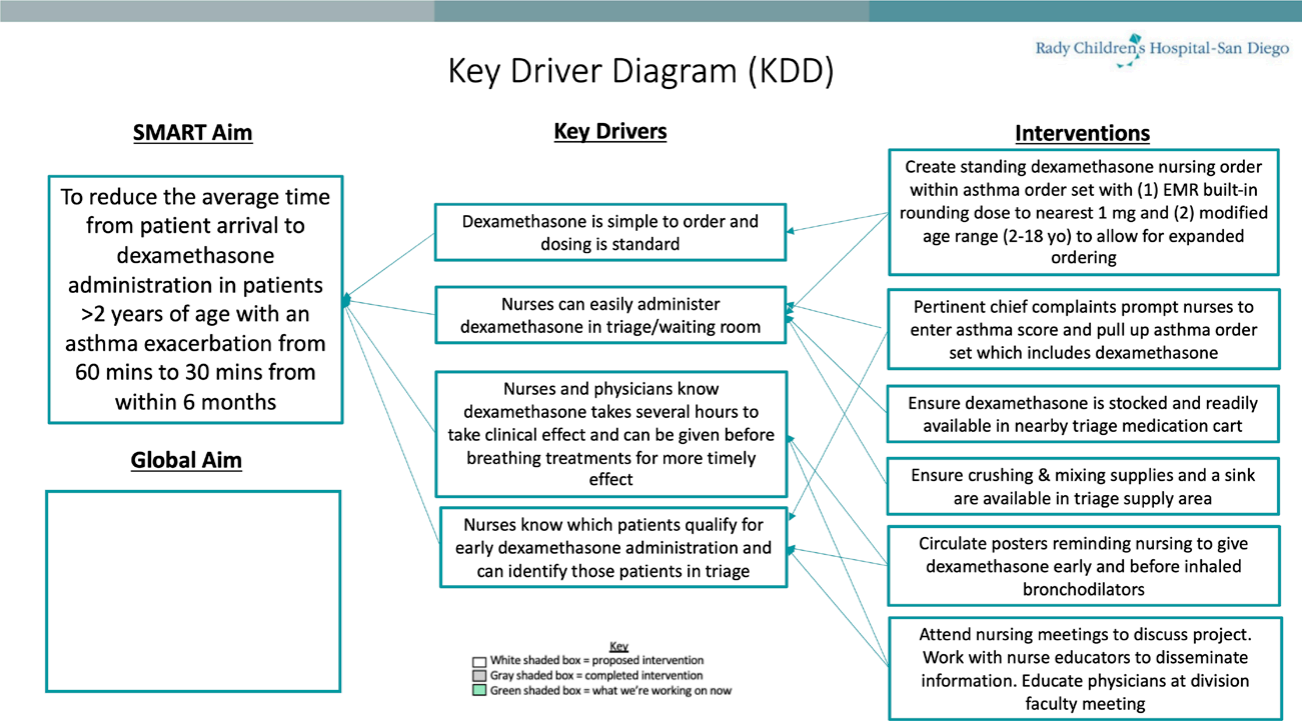
Key Driver Diagram

A review of data 6 months before the initiative established a baseline mean arrival time to dexamethasone administration of 59 minutes. Subsequently, the goal was to reduce this number by 50% (to 30 minutes) within six months. This was chosen as an achievable goal based on feedback from the multidisciplinary team and interest as to how it may impact secondary process measures, given averages below an hour are rarely reported in the literature. Using Cincinnati Children’s simplified failure mode and effects analysis and Gemba process mapping, the ED QI team determined the project’s key drivers and interventions and constructed a key driver diagram (Fig. 1), which was periodically updated throughout the project. Several themes contributing to delays in dexamethasone administration were identified, including lack of a standing nursing order, lack of medication availability in the triage area, lack of dosage rounding in the electronic medical record (EMR), and concerns amongst staff regarding interrupting bronchodilators to administer dexamethasone.

## INTERVENTIONS

There were three categories of interventions implemented over the study period using Plan-Do-Study-Act cycles: (1) development and EMR integration of a nurse standing dexamethasone order, (2) awareness and education of the QI initiative, and (3) provision of necessary supplies to execute interventions.

### Standing Dexamethasone Order

As part of the initial interventions in October 2021, a standing preselected order for dexamethasone was created and integrated into an existing asthma order set for nurses, which had previously only included bronchodilators. The order set was also modified for patients aged 2–18 years, whereas previously, it was limited to patients aged ≥6 years. The standing dexamethasone order was automatically rounded to the nearest 1 mg for ease of administration. In March 2022, the EMR was further modified to prompt asthma severity scoring and order set usage during triage assessment for chief complaints, including “asthma exacerbation,” “wheezing,” or “shortness of breath.” Although excluded from our data, these interventions could be applied to first-time wheezing patients using the nursing order set.

### Awareness and Education

In November 2021, education was disseminated to ED nurses at their staff meetings. Educational posters were also displayed in highly trafficked sections of the ED, including the triage area. The posters emphasized the recommendation to not delay dexamethasone in favor of initiating bronchodilator treatments. From October 2021 to March 2022, the lead physician attended nursing staff meetings every quarter to remind nurses about the project, gather feedback about existing interventions, and solicit suggestions for additional interventions that could be made to improve the timely administration of dexamethasone. Additionally, a nursing lead was identified, who provided aggregated feedback from nurses and assisted in planning interventions. PEM physicians were educated about the project at a departmental faculty meeting in October 2021, a month after the project was presented to the ED Quality Assurance Performance Committee for feedback and awareness.

### Provision of Supplies

In our ED, dexamethasone is stocked in tablets and routinely crushed and then mixed with syrup for better taste. In December 2021, the medication cabinet in the triage area was stocked with 1-mg tablets of dexamethasone in addition to an existing supply of 4-mg tablets. Crushing and mixing supplies were also provided in the triage area. These changes were initiated based on real-time observations by the authors and feedback from ED triage nurses as the project progressed.

### Measures

The primary outcome measure was the time from ED arrival to dexamethasone administration in patients experiencing an asthma exacerbation. Additional outcome measures included the percentage of patients admitted and the LOS in the ED, as defined by the time discharge procedures were complete.

## ANALYSIS

Microsoft Excel and QI Macros were used to develop statistical process control charts to examine measure changes over time. Established rules for the interpretation of control charts^23^ were used. The centerline and control limits were revised when an intervention was associated with special cause variation, as defined by 8 consecutive points above or below the mean. Special cause variation in the form of a single data point outside the control limits was investigated for possible causes to guide improvement.

## RESULTS

**Fig. 2.**
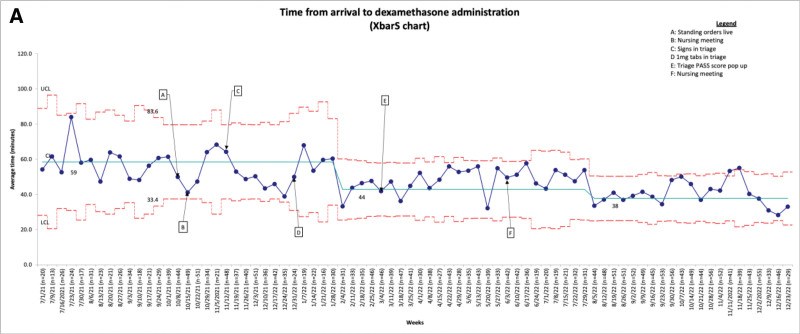
Time from arrival to dexamethasone administration.

Our initiative included 3,192 qualifying patients presenting with asthma exacerbation over 18 months. The average patient age preintervention was 6.4 years (6.1 postproject initiation, *P* value 0.932), and the average initial asthma score was 8.1 (8.4 postproject initiation, *P* value 0.932). Over the 10 months from the project’s start, we achieved two statistical process control chart shifts associated with our interventions. We reduced the time from arrival to dexamethasone administration from 59 to 38 minutes (Fig. 2). This change was sustained for an additional four months from the last shift.

**Fig. 3.**
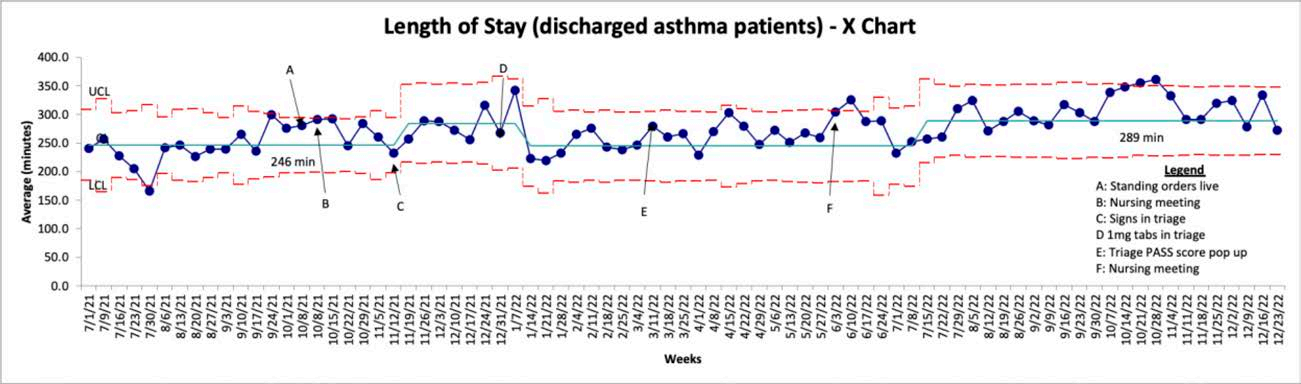
Length of stay (discharged asthma patients).

**Fig. 4.**
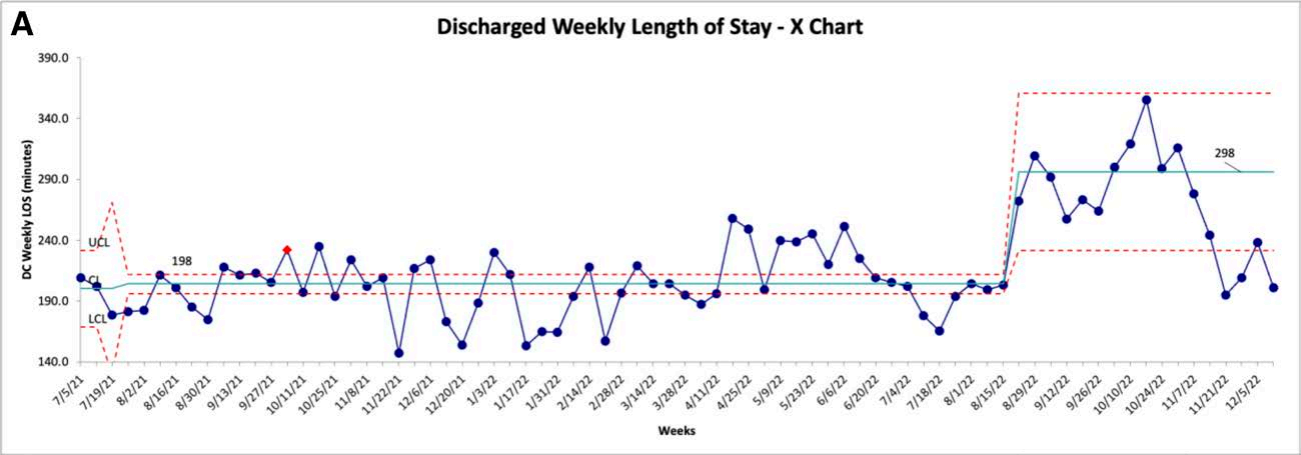
Discharged weekly length of stay.

**Fig. 5.**
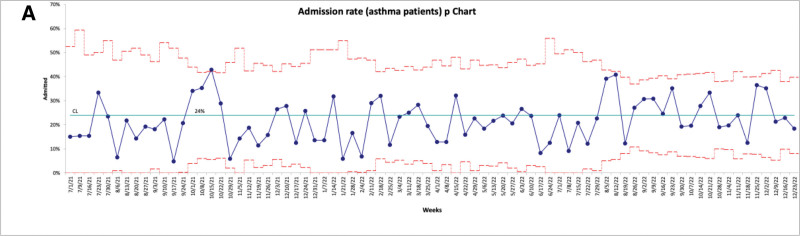
Admission rate (asthma patients).

In July 2022, an increase in the LOS in the ED for discharged patients meeting study inclusion criteria was noticed (Fig. 3). This is thought to be due to special cause variation because it occurred during a time of significantly high patient volumes when the LOS for all discharged patients in the ED increased (Fig. 4). This was likely due to COVID-19 and the effect of quarantine on our viral season, which resulted in inpatient bed shortages and increased ED patient boarding. We also tracked the percentage of patients receiving dexamethasone for asthma exacerbations who were admitted (Fig. 5). We noted no shift throughout the study, with an average admission rate of 20.5%.

We observed rare instances of dexamethasone administration during the study period in the 0- to 2-year age group. However, upon reviewing reported cases, these were found to be in clinical scenarios where administration was appropriate (ie, patients with a history of asthma or wheezing-associated respiratory illnesses responsive to bronchodilators under 2 years of age). No cases of inappropriate dexamethasone administration in this age group were identified during the study period.

## DISCUSSION

In this QI initiative, we successfully reduced the average time from ED arrival to steroid administration for acute asthma exacerbations presenting to our ED by implementing a nurse-driven system for administering oral dexamethasone in triage. Key interventions included creating and implementing a standing electronic dexamethasone order within an existing asthma order set, education of and feedback from key ED staff, including nurses and respiratory therapists, and providing the supplies necessary to administer dexamethasone in the triage area. Our nurse-driven system removes the need for physician evaluation or a physician order before administering dexamethasone to qualifying patients.

Similar systems for nurse-initiated administration of oral steroids and bronchodilators to pediatric patients presenting to the ED with asthma exacerbation have previously been published in the literature.^15,17–19^ One study showed that a nurse-driven clinical pathway for bronchodilator administration led to decreased LOS with no increase in readmission in the inpatient setting.^24^ Implementing respiratory therapist-driven asthma care pathways has been shown to reduce pediatric intensive care unit LOS.^25^ While several prior studies have also demonstrated significant reductions in the average time from ED arrival to steroid administration, our study achieved the lowest reported average time. Additionally, our study population was notably larger.

Although this project reduced the average time to dexamethasone administration, the initiatives and interventions implemented did not have a greater impact on LOS or admission data. Additionally, while the initial goal was to decrease time to steroid administration by 50% in 6 months, 10 months was required to achieve two shifts. Even with this prolonged study period, we did not achieve our goal of reducing the time to dexamethasone administration to 30 minutes, with 38 minutes being our final sustained centerline. This still puts our average time well below 60 minutes, broadly considered the standard of care. We chose 30 minutes as an achievable goal in our QI initiative to reduce the time to dexamethasone administration by 50% and also to be interested in how this may impact secondary outcome measures.

Despite this substantial improvement in time from arrival to dexamethasone administration, the average LOS for patients with asthma discharged from the ED increased throughout the study period. This contrasts Zemek et al, who observed a statistically significant decrease in LOS, whereas Brown et al and Sneller et al did not observe any statistically significant changes in LOS. Although our LOS for discharged patients with asthma did rise over the study period, this increase was lower than the global rise in LOS for all patients discharged from our ED. Overall, ED LOS for discharged patients increased 48% (Fig. 4) during our study period, whereas LOS for discharged patients with asthma only increased 17% (Fig. 3). Thus, it is likely that our interventions positively impacted the average LOS for the study population. These overall LOS increases were thought to be due to changes in ED volume and staffing, as PEDs across the country were experiencing unprecedented volumes and acuity of patients. This result is thought to be due to COVID and resulting quarantines leading to changes in the expected viral seasons, making staffing predictions challenging. In contrast to Sneller et al, we did not observe a significant change in admission rates for the study population. Still, again, our project happened during a time of unusually high patient volume and acuity.

Additional limitations of the study are its confinement to a single hospital and EMR, potentially making it difficult to generalize to other institutions with different EMRs and/or policies or protocols guiding nurse standing orders. Another perceived limitation during the study period was that a significant portion of nursing staff at our institution was categorized as “travelers” (temporarily contracted for three-month intervals). Depending on their contract dates, they may not have participated in the educational interventions described but were still participants in patient care during the project. While potentially limiting, this phenomenon occurs at pediatric hospitals nationwide due to seasonality^26^ and may make our observations generalizable to other institutions. However, despite this possible limitation, we succeeded in decreasing the time to oral corticosteroids.

The lack of a balancing measure is another limitation. During the project’s planning phase, we set out to track inappropriate administration of dexamethasone as a balancing measure. We defined this as patients aged under 2 years receiving dexamethasone via the nursing order set, given there is diagnostic uncertainty with wheezing in this age group (eg, bronchiolitis-associated wheezing). We could not track this due to an overlap with a standing nursing order of dexamethasone for croup. However, we only observed rare instances of dexamethasone administration in the 0- to 2-year age group during the study period. A review of reported cases found these to be clinical scenarios where the administration was appropriate (ie, patients with a history of asthma or wheezing-associated respiratory illnesses responsive to bronchodilators under two years of age). Additionally, no significant increase in triage time was observed by nursing leadership as a result of our interventions, though data on triage time are not available.

The next steps for this project will be targeted at ensuring sustainability. Our study demonstrated potential for longevity with continued results six months after our last intervention. However, further observation is needed to ensure continued results through high seasonal ED volume periods.

This project demonstrated a successful expansion of nurse-driven medication administration. Further refinement and emphasis on triage medication administration may, in the appropriate clinical context, be replicated and incorporated into other QI initiatives in the future, resulting in enhanced clinical care and outcomes for a broader category of patients.

## CONCLUSIONS

Using QI methodology in a high-volume tertiary PED, we successfully decreased the time from arrival to dexamethasone administration from 59 to 38 minutes over 10 months. We sustained this change for an additional 4 months despite an increased LOS for all ED patients.

## ACKNOWLEDGMENT

Special thank you to Jasmine Miller-Berrios, RN, Cynthia Sepulveda, ED QAPI Committee, Epic ASAP Team.
